# An Anisotropic Microstructure Evolution in a Solid Oxide Fuel Cell Anode

**DOI:** 10.1186/s11671-019-3226-1

**Published:** 2020-01-03

**Authors:** Grzegorz Brus, Hiroshi Iwai, Janusz S. Szmyd

**Affiliations:** 1 Department of Fundamental Research in Energy Engineering, AGH University of Science and Technology, 30 Mickiewicza Ave., Krakow, 30-059 Poland; 2 Department of Mechanical Engineering and Science, Kyoto University, Nishikyo-ku, Kyoto, 615-8540 Japan

**Keywords:** Fuel cells, Nanotomography, Tortuosity, Microstructure

## Abstract

The presented research shows that the long-term operation of a solid oxide fuel cell can lead to substantial anisotropic changes in anode material. The morphology of microstructure in the investigated stack was observed before and after the aging test using electron nanotomography. The microstructural parameters were estimated based on the obtained digital representation of the anode microstructure. Anisotropy was discovered in two of the three phases that constitute the anode, namely nickel and pores. The third component of the anode, which is yttrium-stabilized zirconia, remains isotropic. The changes appear at the microscale and significantly affect the transport phenomena of electrons and gasses. The obtained results indicate that the reference anode material that represents the microstructure before the aging test has isotropic properties which evolve toward strong anisotropy after 3800 h of constant operation. The presented findings are crucial for a credible numerical simulation of solid oxide fuel cells. They indicate that all homogeneous models must adequately account for the microstructure parameters that define the anisotropy of transport phenomena, especially if microstructural data is taken from a post-operational anode.

## Background

**Fig. 1:**
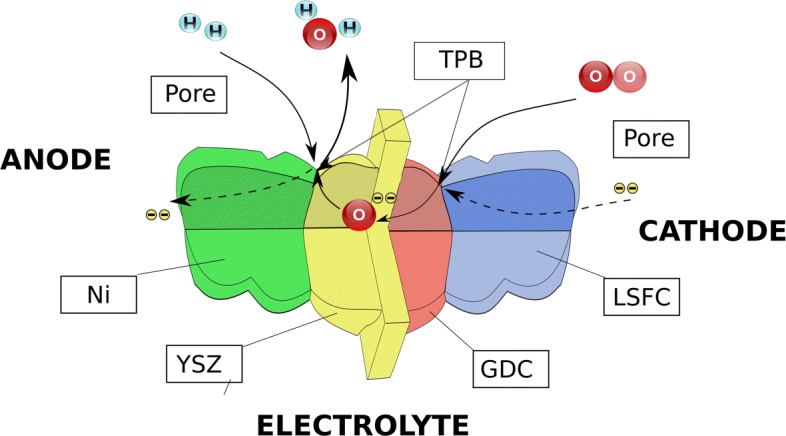
A schematic view of the transport phenomena across a typical solid oxide fuel cell with a highlighted role of the microstructure

A solid oxide fuel cell (SOFC) is an electrochemical device that converts the chemical energy of hydrogen directly into electricity. A single cell usually has a form of a flat plate in which an impervious and dense ion-conducting electrolyte is sandwiched between two porous catalytic electrodes: an anode and a cathode. Fuel is fed to the anode side, and the air is supplied to the cathode. The gasses cannot mix to avoid unproductive combustion. Instead, gasses hit catalyst material, lose their electrons, and form capacitors on both sides of an electrolyte. Because the reaction is slower on the cathode side, a potential difference appears between the two electrodes. This potential difference, together with an oxygen pressure gradient, is a driving force that moves oxygen ions from the cathode to the anode. In this respect, the morphology of the electrode microstructure is of crucial importance. A typical anode consists of a nickel phase (Ni), an yttria-stabilized zirconia phase (YSZ), and a pore phase. Each material plays an essential role in the transport processes across the SOFC by providing a pathway for different species. In the case of an anode, the YSZ phase provides pathways to oxygen ions, the Ni phase for electrons, and the pore phase allows gasses to penetrate the electrode. The electrochemical reaction can occur only at the line where all three phases are in contact, the so-called triple phases boundary (TPB). The transport phenomena across the cell are presented schematically in Fig. 1 [1].

Because of the complexity of the anode composite, the microstructure-oriented design becomes a crucial step in SOFC development [2–7]. In this work, we investigate the microstructure changes that occur in a solid oxide fuel cell anode over a long period of operation. To provide in-depth analysis, we focus on the anisotropic tortuosity factor that reflects the complexity of microstructure in a given direction. The structure analysis is conducted using a scanning electron microscope coupled with the focused ion beam. This technique was introduced to the field of SOFC in 2006 by Wilson et al. [8]. The method allows the direct observation of many following sections and converts the results to a 3D digital representation of microstructure. From the reconstructed microstructure, it is possible to evaluate the microstructure parameters [9–11]. These parameters, as directly obtained from the real electrode structure, are of key importance to understand the evolution of anode microstructure during the long run of a fuel cell system. This technique was widely used to improve numerical modeling [12–17] and more recently to understand the degradation mechanisms of a solid oxide fuel cell anode [18–24]. That became a valuable endorsement to the recent crystallographic studies [25, 26].

In this paper, we report, for the first time, the anisotropic character of microstructure evolution during the long-term operation of a SOFC stack. We show that the microstructure evolves mainly due to anisotropic migration, growth, and coarsening of nickel particles.

## Experimental Apertures

### Modular Stack Testing Bench

**Fig. 2:**
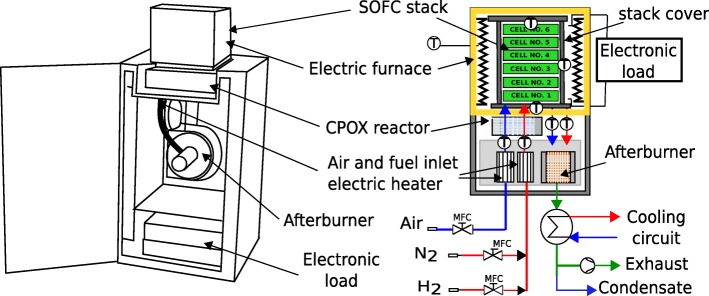
A schematic view on the Modular Stack Test Bench

The aging test was conducted using a Modular Stack Test Bench (MSTB) designed and developed by SOLID Power, a leading European SOFC manufacturer. The schematic view on the set-up is presented in Fig. 2. The stack is located inside an electric furnace.

The fuel and air are supplied to the system via mass flow controllers and preheaters. Both air and fuel are fed into the catalytic partial oxidation (CPOX) reactor (if the fuel contains methane) or bypasses it if the fuel is a mixture of hydrogen and nitrogen. Air is supplied to the cathode channel after preheating. Part of the oxygen in the fed air is consumed in the electrochemical reaction. At the same time, air is used to remove heat from the cell stack. Then, air is delivered to the afterburner to combust the unused fuel from the anode channel. On the other side of the air processing, the fuel is oxidized to generate electricity. The fuel which in this study was the mixture of hydrogen (H_2_) and nitrogen (N_2_) is fed to the anode channel after preheating. The residual fuel is oxidized in the afterburner. After the combustion process, the gas is cooled down, the condensed water is separated, and the dry gas is finally exhausted to the ambient air.

Seven thermocouples marked by “T” in Fig. 2, monitor the temperature distribution. Each bipolar plate is connected to a wire, which is then connected to a potentiostat. This configuration allows for obtaining the current-voltage characteristics for every cell in the stack. The dimensions of a cell are 60 × 80 [mm × mm]. The active cell area available for the reaction is 48 [cm^2^]. The cell performs fuel utilization of up to 75% and can achieve high power density larger than 1 [W cm ^−2^]. The stack is organized in a co-flow configuration where fuel and air flow is in the same direction. A mixture of hydrogen and nitrogen was used as a fuel in the system.

More details about the set-up can be found elsewhere [27, 28].

### Focused Ion Beam–Scanning Electron Microscope

A dual beam system combines a scanning electron microscope (SEM) and a source of focused gallium ions beam (FIB) in one chamber. The SEM is used for imaging and the FIB serves mostly for milling. For a limited situation, FIB can be used for the observation. The system gives the unique possibility of subsequent sections of an investigated sample. The usual size of the sample of material that can be located in holder is 25 mm^2^. The FIB-SEM set-up and the measuring procedure are schematically presented in Fig. 3. The ion gun direction is perpendicular to the sample surface, and the electron gun is tilted at some angle with respect to the source of the ions to allow for the observation of a sample’s section. The volume of interest from the sample that can be observed during a single procedure is about 1000 *μ*m^3^. The focused ion beam is used to fabricate a trench and expose a sample’s intersection that meets the requirements for the minimal representative volume size. After the trench is made, the intersection is polished using low energy Ga ^+^ beam and the image is taken using an in-lens detector. This yields to exceptionally good contrast between the investigated phases: Ni, YSZ, and pore. After a SEM image is taken, the FIB gun mills to expose another intersection, and another layer of material is removed. The “cut-and-see” procedure is repeated until obtaining 200-300 images depending on the volume of interest. This procedure is known as the sectioning. The procedure can be summarised in the following steps:

1. A carbon layer is deposited on the volume of interest.

**Fig. 3:**
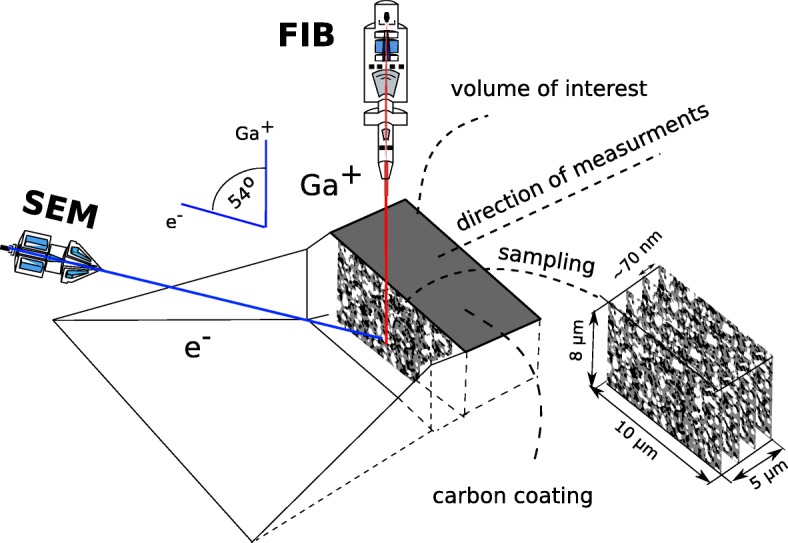
The configuration of a dual beam system
2. A trench is fabricated to give access to the sample’s intersection.
3. The cross section is polished with the low current beam.
4. A SEM picture of an observed intersection is taken using an in-lens secondary electron detector.
5. A FIB gun uses a beam of Ga+ ions to mill into the sample to expose another intersection.
6. A “cut and see” procedure is repeated to obtain the sequence of 2D images.

The idea of the cut-and-see procedure is presented in Fig. 3.

## Experimental Methodology

**Fig. 4:**
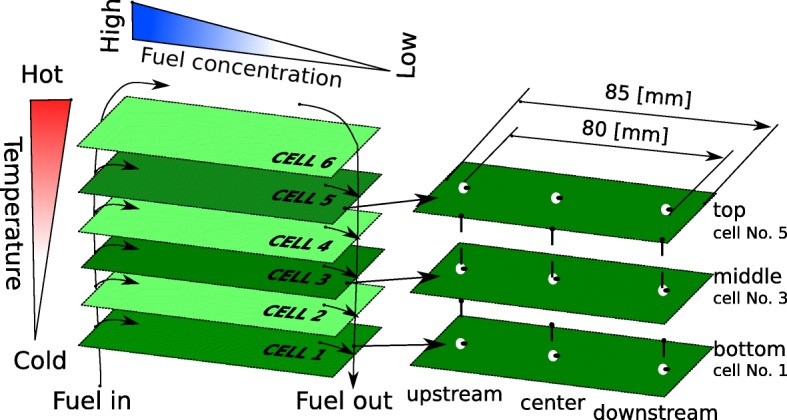
The locations of the selected samples in a cell and in the stack

The presented study was divided into two separate parts: a power generation experiment and a microstructural study. The endurance study was conducted by keeping the stack under constant load over an extended period. To reduce the duration of the test, the temperature was elevated up to 800 ^*o*^C and the imposed current was 19.4 A to provide 90 W of output power at the beginning of the experiment. The fuel utilization factor was 75%. The detailed experimental conditions are summarized in Table 1. After the aging test, the stack was disassembled and nine samples were selected for the post-test microstructural analysis. Three samples were extracted from the cells no. 1, 3, and 5 (located at the upstream, center, and downstream of each cell) as presented in Fig. 4. An additional cell, the so-called reference cell, was a new cell just after the reduction process. The cell was provided by the manufacturer and did not participate in the electrochemical tests. Therefore, it is reasonable to assume that the microstructure of the reference cell represents the microstructure from before the aging test. All samples for FIB-SEM analysis had a form of 5 mm × 5 mm squares and were cut off from the cell (6 cm × 8 cm) using a diamond pen. Prior to the microstructural studies, all samples were impregnated with epoxy resin and polished with sandpaper. The impregnation is important for the recognition of the pore phase during SEM imaging. All nine samples were analyzed using the FIB-SEM technique.

**Table 1:** The long-term operation experiment conditions

Controlled process parameters	Value
Hydrogen flow rate	1.8 L min ^−1^
Nitrogen flow rate	1.2 L min ^−1^
Air flow rate	18.0 L min ^−1^
Cell stack electric furnace	800 ^∘^C
Air inlet heater	630 ^∘^C
Fuel inlet heater	550 ^∘^C

**Fig. 5:**
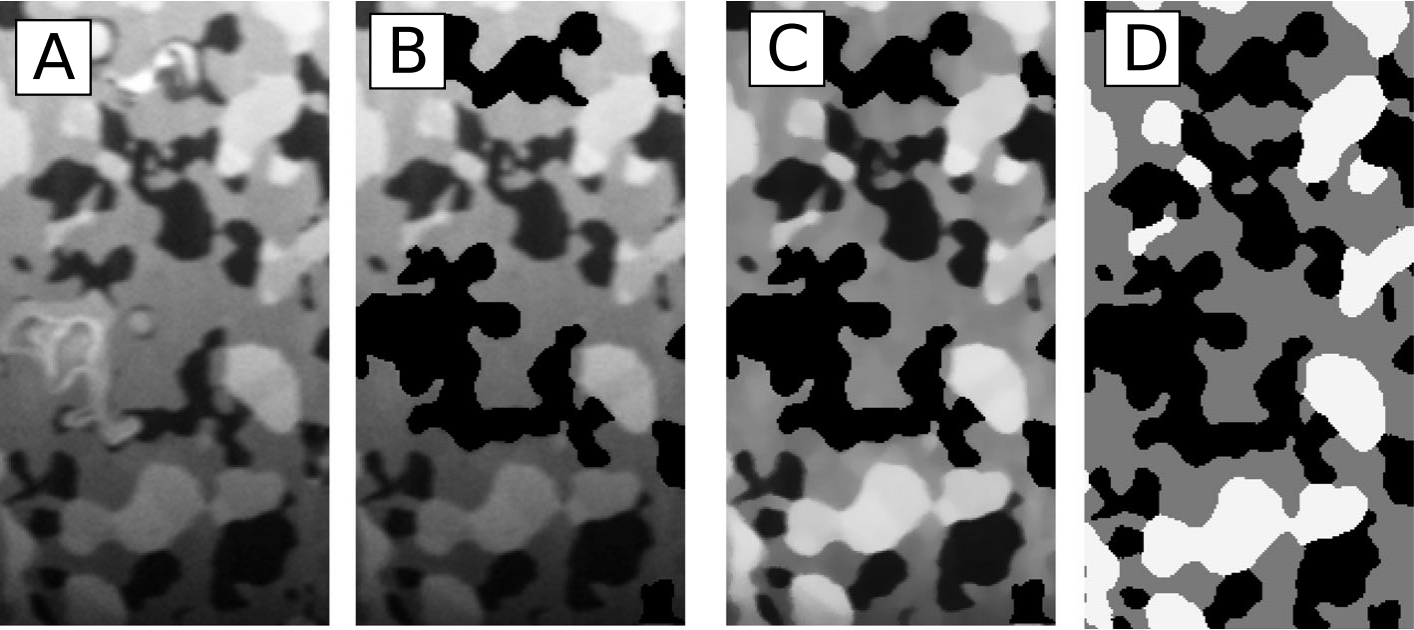
The workflow of the image processing and phase labeling. **a** A raw image. **b** Manually removed experimental artifacts. **c** Filtering. **d** Phase labeling where white represents nickel, black is pores, and gray denotes YSZ

The set of SEM images obtained for each sample underwent the image segmentation process to assign one of three phases to each region of the SEM image. The segmentation is a process of labeling the image regions based on its brightness, which conducted semi-automatically, required up to one month of an operator work per sample. After succeeding in the image segmentation process, image resampling takes place (see Fig. 5).

**Fig. 6:**
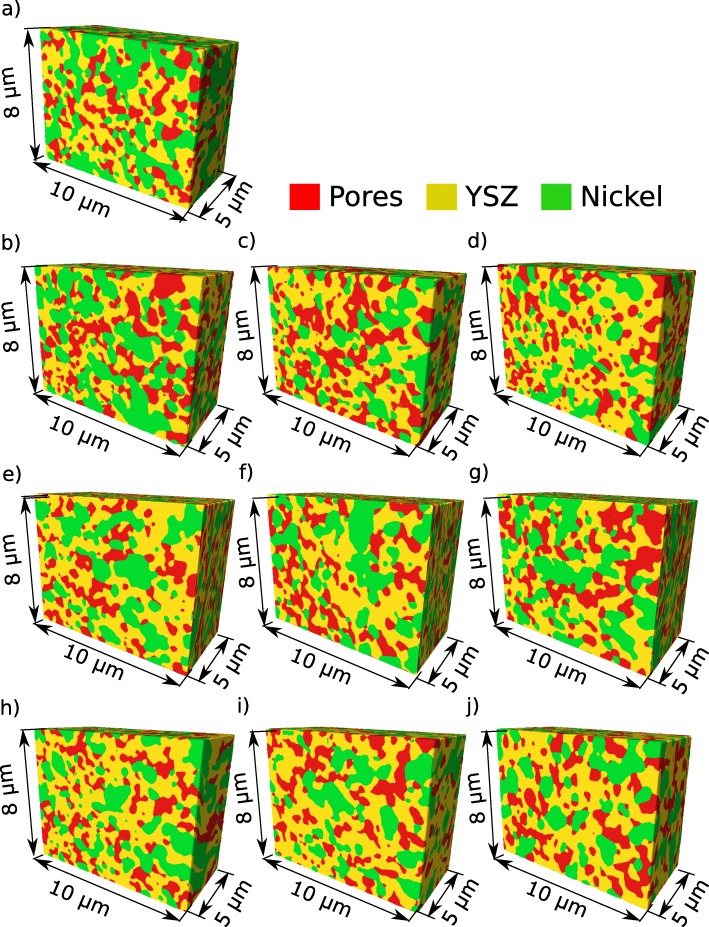
Digital material representation of anode microstructure before and after the aging test. **a** Reference sample. **b** Cell 5 upstream. **c** Cell 5 center **d**. Cell 5 downstream **e** Cell 3 upstream. **f** Cell 3 center **g**. Cell 3 downstream. **h** Cell 1 upstream. **i** Cell 1 center. **j** Cell 1 downstream

Random walk simulation, introduced later in this paper, requires the cubic voxel. It means that the distance between images should be equal to the image’s pixel size. However, more slices indicate more time needed for the segmentation which is practically infeasible. In practice, the distance between images is more significant than the pixel size to save time during the most time-consuming process of the segmentation. Therefore, segmentation is conducted on the cuboid voxels and converted to a cubic voxel during postprocessing. Based on the resampled images, the surfaces that represent the three-dimensional morphology of each phase were generated by a triangular approximation of the interface between the different regions. The triangulation and the resampling were conducted using AVIZO software by ThermoFisher Scientific. The obtained three-dimensional digital material representations are presented in Fig. 6.

The tortuosity factor is a quantitative measure of microstructure complexity. The concept of tortuosity was introduced to porous media study by Carman [29] who studied a flow through a bed of sand. He introduced tortuosity as a factor that takes into account the elongated diffusion path of fluid inside porous media. In his study, he assumed that a porous bed of thickness *L*_*s*_ could be regarded as a bundle of sinuous capillary tubes with a uniform cross section and length *L*_*e*_. Similarly, for the solid oxide fuel cell anode, the tortuosity can be defined as a ratio of the real diffusion path length and electrode thickness. In this simplified system, tortuosity is defined as the ratio of the length of the real diffusion path, *L*_*e*_, to the path in the straight channel case, *L*_*s*_ (anode thickness):

1 $$  \bar{\tau}=\frac{L_{\mathrm{e}}}{L_{\mathrm{s}}}.  $$

It is important to keep in mind the difference between tortuosity and the tortuosity factor. In light of Carman’s formulation, the tortuosity factor (*τ*) is defined as the square of the tortuosity (*τ*=$\bar {\tau }^{2}$), and it is used as an enhancement factor in a mass diffusion equation:

2 $$  D_{i,{\text{eff}}}= \frac{ \varepsilon }{ \tau} D_{i},  $$

where *ε* is the porosity, *D*_*i*_ is the diffusion coefficient of gas spices *i* inside a gas mixture, and *D*_*i*,eff_ is the effective diffusion coefficient taking into account the elongated diffusion path of the fluid inside the porous media.

In a real anode’s microstructure, the fuel paths might be extraordinarily complicated and gas connections paths can create many branches, separate, and rejoin. Therefore, presenting the tortuosity factor as a square of tortuosity is somewhat symbolic and the real relation between tortuosity and the tortuosity factor cannot be calculated using the capillary model. Some groups overcome that problem using the so-called M factor that explicitly combines into one function geometrical tortuosity, percolation factor (*P*), constriction factor (*β*), and phase volume fraction [30]:

3 $$  M= \frac{\left(\phi P \right)^{a} \beta^{b}}{\bar{\tau}^{c}},  $$

where *a*, *b*, and *c* are constants derived from the methodology described in ref. [31]. The constriction factor introduced by Peterson [32] can be understood as a ratio between the bugles and the bottlenecks. A comprehensive review of existing approaches to estimate the tortuosity factors can be found in a review by Tjaden, Brett, and Shearing [33].

Recently, the diffusion-based algorithms are receiving increasing attention since they do not require a constriction factor. This is because the bottlenecks and the bulges are directly taken into account during the simulation of the diffusion process and the measured value is a direct reduction of the diffusion coefficient [34].

One of the most promising methods here is a random walk process, which can statistically calculate the tortuosity factor for non-sorbing particles. In this method, a large number of markers called random walkers are stochastically distributed in the pore phase presented as red volumes in Fig. 6. At every time step, each walker randomly migrates to the neighboring voxels of the same phase. If the voxel selected for the migration belongs to a different phase, the walker stays at the current position and waits for the next time step. While repeating this process, the mean square displacement of random walkers can be calculated:

4 $$ {\begin{aligned}  \langle \chi \left(\vartheta \right)^{2} \rangle= \frac{1}{ n} \sum_{i=1}^{n} \left[ x_{i}\left(\vartheta \right)^{2} - x_{i}\left(0 \right)^{2} + y_{i}\left(\vartheta \right)^{2} - y_{i}\left(0 \right)^{2} + z_{i}\left(\vartheta \right)^{2} - z_{i}\left(0 \right)^{2} \right], \end{aligned}}  $$

where *𝜗* is the dimensionless time of the random walk procedure, and *n* is the number of the random walkers.

The exact solution of the mean square displacement for a lattice walk in a free space is given by [35]:

5 $$  \langle \chi \left(\vartheta \right)^{2} \rangle=6D_{0}t=a^{2} \vartheta,  $$

where *D*_0_ is the diffusion coefficient in a free space [m^2^ s ^−1^], and *t* is time in [s]. The diffusion coefficient from Eq. (5) can be rewritten as a function of time by calculating derivative:

6 $$  D(t)= \frac{1}{6}\frac{{\rm{d}} \langle \chi \left(\vartheta \right)^{2} \rangle}{{\rm{d}}t}.  $$

Because *𝜗* is a function of time *t*, Eq. (6) takes the following form:

7 $$  D(t)= \frac{1}{6}\frac{{\rm{d}} \langle \chi \left(\vartheta \right)^{2} \rangle}{{\rm{d}}\vartheta} \frac{{\rm{d}} \vartheta}{{\rm{d}}t}.  $$

The parts $\frac {\mbox{{d}} \vartheta }{\mbox{{d}}t}$ can be derived from a part of Eq. (5):

8 $$  6D_{0}t=a^{2} \vartheta,  $$

giving

9 $$  \frac{{\rm{d}} \vartheta}{{\rm{d}}t}= \frac{6D_{0}}{a^{2}},  $$

where *a* is the lattice constant of a simple cubic lattice (i.e., the dimension of the FIB-SEM voxel) [nm].

The tortuosity factor *τ* describes a degree of reduction of the mean square displacement in porous media in comparison to free space [34, 36]:

10 $$ \tau= \frac{D_{0}}{ D(t)}.   $$

By combining Eqs. (7) and (10), one arrives at the following formula:

11 $$  \tau= \frac{D_{0}}{ \frac{1}{6}\frac{{\rm{d}} \langle \chi \left(\vartheta \right)^{2} \rangle}{{\rm{d}}\vartheta} \frac{{\rm{d}} \vartheta}{{\rm{d}}t} },  $$

which after further incorporating Eqs. (8) and (10) becomes:

12 $$  \tau= \frac{a^{2}}{ \frac{{\rm{d}} \langle \chi \left(\vartheta \right)^{2} \rangle}{{\rm{d}}\vartheta} }.  $$

When the transport phenomenon is considered in only one direction, the following expression is relevant:

13 $$ {\begin{aligned} \langle x \left(\vartheta \right)^{2} \rangle_{\rm{free}}=\langle y\left(\vartheta \right)^{2} \rangle_{\rm{free}}=\langle z \left(\vartheta \right)^{2} \rangle_{\rm{free}}=\frac{1}{3}\langle r \left(\vartheta \right)^{2} \rangle_{\rm{free}}=\frac{1}{3} a^{2} \vartheta. \end{aligned}}  $$

Therefore, for the estimation of the anisotropic tortuosity factor the Eq. (12) becomes:

14 $$ \tau_{x,y,z}= \frac{a^{2}}{ 3 \left(\frac{{\rm{d}} \langle \chi \left(\vartheta \right)^{2} \rangle}{{\rm{d}}\vartheta} \right) }.  $$

Because the method is based on a statistic, many walkers and large mean-square displacements are required to estimate the tortuosity factor correctly. Eventually, the walkers will leave the computational domain represented by the digital representation of the microstructure. This is, of course, undesirable because the walk cannot continue out of the computational domain. A phase mirroring is used to avoid this issue. When the walker crosses the boundary, it utterly appears in a new domain which is the mirror reflection of the original microstructure reconstruction. Making a full copy of the digital reconstruction whenever the walker crosses the boundary is too heavy for computer memory, and therefore, special programming techniques were applied to conserve hardware resources. The phase mirroring is the major limitation of the method since the calculated tortuosity reflects only the complexity of the investigated volume of interest (not the entire anode).

Based on the anisotropic tortuosities, we introduce the anisotropy factor defined as follows:

15 $$\begin{array}{@{}rcl@{}} \xi &=& \sqrt{ \left(\tau_{x}-\tau_{r} \right)^{2} + \left(\tau_{y}-\tau_{r} \right)^{2} + \left(\tau_{z}-\tau_{r} \right)^{2} }, \end{array} $$

where *τ*_*x*_,*τ*_*y*_, and *τ*_*z*_ are the anisotropic tortuosity factors in *x*, *y*, and *z* directions, respectively, and *τ*_*r*_ is the tortuosity factor calculated for the total displacement of the walker regardless of the direction in which the displacement takes place.

## Results and Discussion

**Fig. 7:**
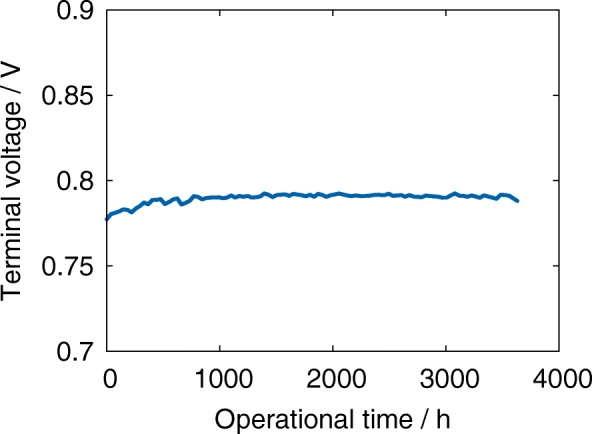
Terminal voltage as a function of operational time during long-term operation

Figure 7 presents an average terminal voltage of the stack during the aging test. As can be seen, there is no evidence of performance reduction. Moreover, the polarization decreases over the first thousand hours of operation. Our previous results indicated that despite the lack of performance deterioration the reaction surface contact surface decreases significantly [24]. We found that the decay of TPB was nonhomogeneous and strongly depended on the location in the stack [24]. In this paper, we show that the evolution of the microstructure is not only inhomogeneous but also anisotropic. The complexity of the anode was estimated based on the anisotropic tortuosity factor derived using the digital material representation presented in Fig. 6.

**Remark** In a real experiment, the diameters of the obtained volume of interest vary from one to another due to the presence of the experimental artifacts. The most common artifacts such as a curtaining effect, shades, and redeposition might limit accessible cross section. As a consequence, the volume that can be recognized appropriately and segmented is different for each measurement. In some cases, we could obtain more than 10 *μ*m in the *z* direction; however, because the curtain affect the sound quality, image in the *y* direction was limited. For others, the image was sharp in the *y* direction, but we could correctly align an only limited number of images. For the quantification, each volume was about 1000 *μ*m^3^. However, just for the sake of the visualization, we trimmed images to one common size of 10 *μ*m × 8 *μ*m × 5 *μ*m to make it possible to juxtapose and compare them in Fig. 6.

**Fig. 8:**
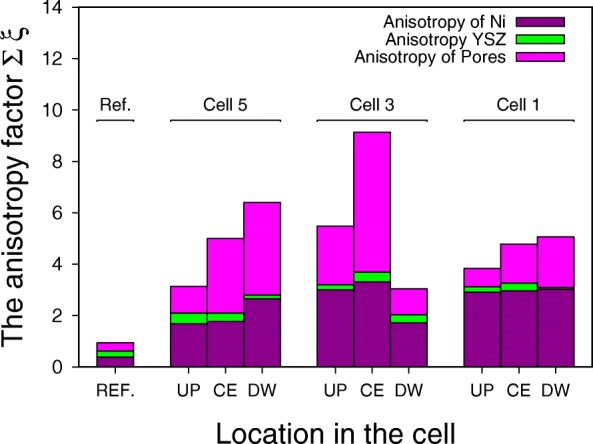
The anisotropy factor at different location in the stack and in a cell, where UP, CE,and DW refers to upstream, center, and downstream of a cell respectively and acronym REF corresponds to the reference cell
- The general tendency is that the anisotropy factor increases as moving downstream the cell.
- Strong anisotropy was observed only for the nickel and pore phases. Yttrium-stabilized zirconium remains isotropic.

The methodology of the tortuosity factor estimation was briefly introduced in section “Experimental Methodology.” Figure 8 shows the anisotropy factors for different locations in the cell and the stack. A comparison with the reference sample is also presented. A common trend observed in the results led us to the following conclusions:

- The reference anode material has isotropic properties which evolve to strong anisotropy during the aging test.

The probable cause of the anisotropy is the nickel particles coarsening [37–39] and migration that was observed in our previous research [23, 24, 40] as well as by other research groups [18, 41]. During the long-term operation, nickel particles migrate from the anode electrolyte interface toward the anode surface. Since the migration occurs mainly in one direction, it leads to the anisotropy of the microstructure. That would also explain why anisotropy affects only the nickel and pore phases. The detailed mechanism is unclear but a possible cause of the nickel particles migration from the anode-electrolyte interface toward the anode surface is the vaporization-deposition of the volatile nickel species such as nickel hydroxide. It will give rise to the inhomogeneity and discontinuous electronic conduction path of nickel [42].

Most gas diffusion models used in the SOFC simulations today assume a homogeneous porous electrode. It is accurate for most of the applications, but our results show this homogeneous assumption may not hold after the degradation. The direct implication of the observation presented in this article is that when one wishes to implement micro-structural parameters of an aged sample into the numerical simulation, it is important to keep in mind what direction of transport phenomena is considered in the model. As a consequence, the proper anisotropic properties from the microstructure parameters should be extracted (if anisotropy is detected). Based on the obtained results, it can be concluded that anisotropy is especially important while diffusion is being considered since the tortuosity factor express quantitatively the rate of reduction of diffusion coefficient. Mind the anisotropic direction while juxtaposing different anodes’ microstructural parameters taken after long-term operation is another practical suggestion steaming for the presented observation.

## General Conclusions

In this paper, we showed for the first time that the long-term operation of SOFC could lead to the anisotropy of microstructure in the anode. The extended power generation experiment was conducted using a short stack. Locally resolved microstructure analysis was performed before and after the aging test using the FIB-SEM nanotomography. The obtained 3D reconstructions of anode microstructure were implemented into a diffusion-based algorithm to calculate the anisotropic tortuosity factor. The results indicate that long-term operation resulted in strong anisotropy in the nickel and pore phases of the investigated anode. The probable cause of the anisotropic properties of the anode after the aging test is migration, growth, and coarsening of the nickel particles.

## Data Availability

The raw and process data required to reproduced those findings cannot be shared at this time as the data forms part of an ongoing study.

## Abbreviations

CPOX: Catalytic partial oxidation
FIB: Focused ion beam
MSTB: Modular Stack Test Bench
SEM: Scanning electron microscope
SOFC: Solid oxide fuel cell
TPB: Triple phase boundary
YSZ: Yttria stabilized zirconia
